# Effect of speaking valve use on decannulation in tracheostomized patients

**DOI:** 10.1097/MD.0000000000050219

**Published:** 2026-08-14

**Authors:** Minghua Yuan, Fengjun Liu, Xia Wu, Haifei Peng, Ting Hu, Xueqin Yu, Xiaoyuan Wang

**Affiliations:** a Rehabilitation Medicine Department, Yichun People’s Hospital, Yichun City, Jiangxi Province, China; b Geriatrics Department, Yichun People’s Hospital, Yichun City, Jiangxi Province, China.

**Keywords:** decannulation, diaphragmatic function, retrospective study, speaking valve, swallowing function, tracheostomy

## Abstract

This study investigates the impact of speaking valve use, in addition to routine treatment and rehabilitation, on decannulation outcomes and related physiological functions in tracheostomized patients. This retrospective cohort study included adult inpatients who underwent tracheostomy from January 2023 to September 2025. Patients were divided into 2 groups based on whether they used a speaking valve. Propensity score matching was applied to control confounding factors, and 80 patients were ultimately included (40 in each group). Baseline data, primary outcomes (decannulation time, success rate, and 72-hour reintubation rate), and secondary outcomes (swallowing function, airway protection, diaphragmatic function, and respiratory physiological parameters) were collected. After matching, baseline characteristics were balanced between the 2 groups (all *P* > .05). The speaking valve group had a shorter time to decannulation than the nonspeaking valve group (15.5 [11.0–21.0] days vs 21.0 [16.0–28.5] days; unadjusted *P* = .008, Bonferroni-adjusted *P* = .024). The speaking valve group also showed a numerically higher successful decannulation rate (85.0% vs 62.5%; unadjusted *P* = .034, adjusted *P* = .102) and a numerically lower 72-hour reintubation rate (7.5% vs 25.0%; unadjusted *P* = .041, adjusted *P* = .123), although these differences did not remain statistically significant after Bonferroni correction. Secondary outcomes showed that the speaking valve group demonstrated greater improvements in swallowing function, airway protection, diaphragmatic function, and respiratory physiological parameters. In tracheostomized patients, speaking valve use was associated with a shorter time to decannulation and improvements in swallowing function, airway protection, diaphragmatic function, and respiratory physiology. Favorable numerical trends were also observed in successful decannulation and 72-hour reintubation rates, although these differences were not statistically significant after correction for multiple comparisons. Prospective randomized studies are needed to confirm whether speaking valve use improves these clinical outcomes.

## 1. Introduction

Tracheostomy is a critical life-support intervention frequently used in patients requiring prolonged mechanical ventilation, upper airway protection, or secretion management.^[1,2]^ Although it effectively secures the airway, tracheostomy disrupts normal upper airway physiology, resulting in loss of phonation, impaired swallowing, reduced cough effectiveness, and compromised airway protection. These dysfunctions often persist even after stabilization of the primary disease, becoming important barriers to successful decannulation.^[3,4]^ Because decannulation represents the recovery of integrated respiratory and airway protective function, interventions that actively facilitate restoration of these physiological mechanisms may shorten decannulation time and improve patient outcomes. However, conventional management strategies primarily focus on respiratory support and infection control, while targeted approaches aimed at restoring upper airway function remain limited.^[5,6]^

Against this clinical background, the speaking valve – an easy-to-use 1-way valve device – has demonstrated unique therapeutic value. When attached to the tracheostomy tube opening, it allows air to enter through the tube during inhalation while directing exhaled airflow upward through the glottis and out of the mouth and nose, thereby partially restoring physiological airflow through the upper airway.^[7]^ This mechanism not only enables patients to regain phonation immediately – which profoundly benefits psychological well-being and communication – but, more importantly, may promote physiological recovery through multiple pathways: restoring subglottic pressure and laryngeal sensory feedback may improve swallowing coordination; reestablishing expiratory glottic closure may enhance cough effectiveness; and restoring physiological end-expiratory pressure may improve oxygenation and reduce diaphragmatic load.^[8,9]^

Despite the promising theoretical mechanisms and the positive clinical effects observed with speaking valve use, significant gaps remain in the current body of evidence. Most existing studies are limited in sample size, lack methodological rigor, and focus primarily on subjective patient experiences, phonation ability, or improvements in single physiological parameters.^[10]^ High-quality evidence demonstrating whether the use of a speaking valve can truly translate into improvements in hard clinical endpoints – such as increased decannulation success rates, reduced time to decannulation, and decreased reintubation rates – remains insufficient. In particular, there is a lack of clinical studies that adequately control for confounding factors and systematically evaluate the device’s integrated effects on multiple physiological systems.

Therefore, this study aims to conduct a retrospective cohort analysis using propensity score matching (PSM) to minimize potential confounding bias and to comprehensively assess the impact of speaking valve use on decannulation outcomes in tracheostomized patients. In addition to examining key clinical endpoints such as decannulation success rate, time to decannulation, and reintubation rate, we will also evaluate objective improvements in swallowing function, airway protection, diaphragmatic performance, and respiratory physiological parameters. Our goal is to provide more comprehensive and reliable evidence regarding the clinical value of speaking valve therapy and to offer new insights for optimizing the holistic management of tracheostomized patients.

## 2. Materials and methods

### 2.1. Study design

This study was approved by the Ethics Committee of Yichun People’s Hospital. This retrospective cohort study included adult patients who were hospitalized in our institution between January 2023 and September 2025, underwent tracheostomy, and met the criteria for decannulation assessment. According to whether they used a speaking valve before decannulation, patients were divided into 2 groups:

Speaking valve group (exposed group): patients who wore a speaking valve during the tracheostomy period as part of routine clinical care and had complete related documentation.

Nonspeaking valve group (nonexposed group): patients who underwent tracheostomy during the same period but did not use a speaking valve.

To eliminate the impact of confounding factors, PSM was applied, and a total of 80 patients with matched baseline characteristics were ultimately obtained, with 40 patients in each group.

### 2.2. Inclusion and exclusion criteria

Inclusion criteria: patients who were hospitalized in our institution and underwent tracheostomy between January 2023 and September 2025; age ≥ 18 years; entered the routine respiratory management pathway after tracheostomy and underwent decannulation readiness assessment; had complete and traceable clinical data, including tracheostomy date, decannulation assessment records, and clear documentation of speaking valve use; reached a clinically assessable condition prior to decannulation (e.g., stable spontaneous breathing, observable airway reflexes, not under deep sedation/paralysis); had clearly documented decannulation outcomes (success, failure, or reintubation with complete records).

Exclusion criteria: missing or incomplete medical records that made it impossible to determine speaking valve use or decannulation outcomes; patients who died, left the hospital against medical advice, or were transferred prior to decannulation assessment due to deterioration; persistent deep consciousness impairment, coma, or inability to cooperate with evaluation at the time of decannulation; concomitant severe upper airway structural abnormalities (e.g., severe laryngeal stenosis, vocal cord fixation, cervical tumors) that significantly affected decannulation feasibility or speaking valve safety; clear contraindications to speaking valve use before decannulation (such as high airway resistance, severe secretion retention, inability to tolerate respiratory load), resulting in an inability to perform effective evaluation; non-standardized decannulation pathways due to special treatment protocols or individualized clinical plans, making comparison with routine procedures impossible.

### 2.3. Treatment methods

#### 2.3.1. Basic treatment protocol

All patients included in the study, regardless of group assignment, received multidisciplinary comprehensive treatment based on the standardized clinical pathway of our institution. The protocol was jointly implemented by attending physicians, rehabilitation therapists, and specialized nursing staff. It covered mechanical ventilation management, fluid and nutritional support, infection prevention and control, organ function monitoring and support, and routine physical therapy. The goal was to create optimal physiological conditions for successful decannulation.

#### 2.3.2. Routine physical therapy

Both groups received systematic rehabilitation training. The training program included postural management, range-of-motion exercises, and muscle-strengthening exercises. Postural management began with elevating the head of the bed to 30°, gradually progressing to 90° sitting, bedside sitting, and sitting out of bed depending on patient tolerance. Each session lasted 10 to 30 minutes and was conducted twice daily. Range-of-motion exercises were performed first as passive full-range movements by the therapist, then progressed to assisted active and active exercises based on recovery. These were conducted in 2 to 3 sets per day, with 10 to 30 repetitions per set, 3 to 5 d/wk. Muscle-strengthening exercises followed a progressive approach, starting with assisted exercises and advancing to active and resistance training, with the same frequency as range-of-motion exercises.

#### 2.3.3. Intervention protocol for the speaking valve group

Patients in the speaking valve group received speaking valve application and training in addition to standard treatment and rehabilitation. A uniformly manufactured speaking valve from Shanghai was used in this study. Before use, medical staff explained the procedure to the patient and obtained consent, and nasogastric feeding was paused 1 to 2 hours in advance. During the procedure, airway and oral–nasal secretions were cleared, the cuff was deflated, and the tube was secured before gently attaching the valve. After installation, patient tolerance was first observed; once confirmed, speech training was initiated, progressing from phonation and counting to words and short sentences. Initial wearing time was 10 minutes, then gradually extended based on patient status. If stable after 1 week, the valve could be worn for prolonged periods during waking hours. Throughout the wearing process, vital signs and subjective sensations were closely monitored to ensure respiratory safety.

Throughout the intervention period, patients exhibited variability in daily wearing duration and progression pace based on individual tolerance, physiological stability, and clinical judgment. While the protocol specified gradual extension of wearing time, some patients experienced tolerance-related interruptions (e.g., transient oxygen desaturation, increased respiratory effort, or excessive secretions) that temporarily delayed progression. These variations were documented in the medical records and reflect the real-world nature of the retrospective data. Notably, all patients in the speaking valve group successfully achieved extended daytime wearing prior to decannulation, ensuring that the post-intervention assessments captured the functional status after adequate adaptation.

### 2.4. Data collection

This study collected data from eligible patients by retrospectively retrieving information from the hospital’s electronic medical record system. All data were collected by trained research personnel and cross-checked after entry to ensure accuracy and completeness. The collected information included the following:

#### 2.4.1. Baseline data

Demographic information, basic clinical characteristics, and disease severity indicators were collected for all patients. These included age, sex, primary diagnosis, duration of mechanical ventilation before tracheostomy, Acute Physiology and Chronic Health Evaluation II score at admission, Glasgow Coma Scale score at the time of tracheostomy, and major comorbidities such as hypertension and diabetes. These data were used to describe the fundamental characteristics of the study cohort and served as covariates for PSM.

#### 2.4.2. Primary outcome measures

The primary outcome measures were decannulation-related results. Data collected included the date of tracheostomy, date of decannulation assessment, decannulation outcome (success or failure), and whether reintubation occurred within 72 hours after decannulation. The time interval (in days) from tracheostomy to decannulation was calculated based on these dates. Decannulation success was defined as remaining free of reintubation or repeat tracheostomy for more than 48 hours after tube removal.

#### 2.4.3. Secondary outcome measures

Secondary outcomes covered multiple functional domains, including swallowing function, airway protection, and diaphragmatic function. To evaluate changes over the course of treatment, data were collected at 2 standardized time points for all patients: at the initial decannulation readiness assessment (baseline, pre-intervention) and at the final assessment immediately prior to decannulation (post-intervention). All assessments were performed by trained research personnel following standardized protocols, and evaluations for each patient were consistently conducted at the same time of day (between 8:00 and 10:00 am) to minimize diurnal variation.

Swallowing function was assessed using the Functional Oral Intake Scale (FOIS) and the Gugging Swallowing Screen (GUSS). FOIS evaluated actual oral intake ability, while GUSS assessed swallowing safety and effectiveness. Airway protection ability was evaluated through both instrumental measurements and clinical observations: peak cough flow was measured using a peak flow meter; cough strength was assessed using the Semi-Quantitative Cough Strength Score (SCSS, 0–5 points). If volitional cough scored 0, cough response to tracheal carina stimulation via catheter was assessed and scored accordingly. Additionally, the daily number of suctioning episodes was recorded by nursing staff over the preceding 24-hour period. Diaphragmatic function was assessed by bedside ultrasound, performed by trained physicians following standard procedures to measure end-expiratory and end-inspiratory diaphragm thickness, and diaphragm thickening fraction was calculated. The Clinical Pulmonary Infection Score was also collected to evaluate the severity of pulmonary infection. However, due to incomplete documentation in a subset of patients and limited variability within the matched cohort, Clinical Pulmonary Infection Score was not included in the final comparative analysis.

#### 2.4.4. Respiratory physiology and clinical stability parameters

Physiological parameters were extracted from nursing charts and the laboratory information system, corresponding to the same 2 standardized time points as the secondary outcome measures (baseline and pre-decannulation). All parameters were obtained with the patient in a supine position, with the head of the bed elevated to 30° after at least 10 minutes of rest. These included peripheral oxygen saturation (SpO_2_), the ratio of arterial oxygen pressure to inspired oxygen fraction (PaO_2_/FiO_2_), respiratory rate, heart rate, and mean arterial pressure. The occurrence of respiratory distress during the pre-decannulation observation period was also documented, defined as new or worsening dyspnea, use of accessory respiratory muscles, paradoxical breathing, or new-onset tachycardia accompanied by agitation.

### 2.5. Statistical analysis

Statistical analysis was performed using SPSS version 26.0. To control for confounding bias, PSM was conducted at a 1:1 ratio using a caliper width of 0.02, yielding 80 matched patients. Continuous variables were expressed as mean ± standard deviation or median (interquartile range), as appropriate, and between-group comparisons were performed using the independent-samples t-test or Mann–Whitney U test. Categorical variables were expressed as number (percentage) and were compared using the chi-square test or Fisher’s exact test, as appropriate.

For the 3 decannulation outcomes reported in Table 2 - time to decannulation, successful decannulation, and reintubation within 72 hours – a Bonferroni correction was applied to account for multiple comparisons. The Bonferroni-adjusted significance threshold was 0.05/3 = 0.0167. Both unadjusted and Bonferroni-adjusted P values are reported in Table 2. For all other analyses, a 2-sided *P* value < 0.05 was considered statistically significant.

## 3. Results

### 3.1. Patient enrollment and baseline characteristics

A total of 80 tracheostomized patients were included in this study. After PSM, 40 patients were assigned to the speaking valve group and 40 to the nonspeaking valve group. There were no statistically significant differences between the 2 groups in terms of age, sex, primary disease distribution, duration of mechanical ventilation before tracheostomy, disease severity scores (Acute Physiology and Chronic Health Evaluation II, Glasgow Coma Scale), or comorbidities (all *P* > .05). The baseline characteristics of the 2 groups were therefore comparable (Table 1).

**Table 1:** Patient selection and baseline characteristics (after propensity score matching, n = 80).

Characteristic	Speaking valve group (n = 40)	Nonspeaking valve group (n = 40)	*P* value
Demographics			
Age (yr), mean ± SD	65.2 ± 10.5	66.8 ± 11.3	.512
Male, n (%)	25 (62.5%)	23 (57.5%)	.818
Primary diagnosis, n (%)			
Stroke	18 (45.0%)	16 (40.0%)	.822
Trauma (brain/polytrauma)	10 (25.0%)	12 (30.0%)	.796
Pulmonary infection/COPD	8 (20.0%)	7 (17.5%)	.952
Postoperative complications	4 (10.0%)	5 (12.5%)	.931
Disease severity			
Duration of Mechanical Ventilation before Tracheostomy (d), median (IQR)	12.5 (8.0–18.0)	11.0 (7.0–17.3)	.436
APACHE II Score (at admission), mean ± SD	18.3 ± 4.1	19.0 ± 4.5	.467
GCS Score (at tracheostomy), median (IQR)	9 (8–11)	9 (7–10)	.389
Comorbidities, n (%)			
Hypertension	22 (55.0%)	20 (50.0%)	.822
Diabetes mellitus	11 (27.5%)	13 (32.5%)	.804

APACHE II = Acute Physiology and Chronic Health Evaluation II, COPD = chronic obstructive pulmonary disease, GCS = Glasgow Coma Scale, IQR = interquartile range, SD = standard deviation.

### 3.2. Comparison of primary decannulation outcomes

The speaking valve group had a shorter median time from tracheostomy to decannulation than the nonspeaking valve group (15.5 [11.0–21.0] days vs 21.0 [16.0–28.5] days). This difference remained statistically significant after Bonferroni correction for the 3 decannulation outcomes reported in Table 2 (unadjusted *P* = .008; Bonferroni-adjusted *P* = .024).

**Table 2:** Comparison of decannulation outcomes With Bonferroni correction.

Outcome	Speaking valve group (n = 40)	Nonspeaking valve group (n = 40)	Unadjusted *P* value	Bonferroni-adjusted *P* value
Time to decannulation (d), median (IQR)	15.5 (11.0–21.0)	21.0 (16.0–28.5)	0.008	**0.024**
Successful decannulation, n (%)	34 (85.0)	25 (62.5)	0.034	0.102
Reintubation within 72 h, n (%)	3 (7.5)	10 (25.0)	0.041	0.123

Bonferroni-adjusted *P* values were calculated for the 3 decannulation outcomes by multiplying each unadjusted *P* value by 3. Statistical significance was defined as an adjusted *P* value < .05.

Bold indicates *P* <.05.

IQR = interquartile range.

The successful decannulation rate was numerically higher in the speaking valve group than in the nonspeaking valve group (85.0% [34/40] vs 62.5% [25/40]), but the difference did not remain statistically significant after Bonferroni correction (unadjusted *P* = .034; adjusted *P* = .102). Similarly, the 72-hour reintubation rate was numerically lower in the speaking valve group (7.5% [3/40] vs 25.0% [10/40]), but this difference was not statistically significant after correction (unadjusted *P* = .041; adjusted *P* = .123; Table 2).

### 3.3. Improvement in swallowing function

Regarding swallowing function, the speaking valve group showed significantly greater improvement. There were no significant differences in FOIS or GUSS scores between the 2 groups before intervention (*P* > .05). After intervention, the improvement in FOIS scores was significantly greater in the speaking valve group (+2.0 [1.0–2.0] vs +1.0 [0.0–1.0], *P* < .001). Similarly, improvements in GUSS scores were more pronounced in the speaking valve group (+5.7 ± 2.5 vs +1.9 ± 1.8, *P* < .001; Table 3).

**Table 3:** Comparison of swallowing function improvement between the 2 groups.

Swallowing outcome measure	Speaking valve group (n = 40)	Nonspeaking valve group (n = 40)	Between-group *P* value
FOIS Score, median (IQR)			
Before intervention	3 (2–4)	3 (2–4)	.85
After intervention	5 (4–6)	4 (3–4)	<.001
Change in score	+2.0 (1.0–2.0)	+1.0 (0.0–1.0)	<.001
GUSS Score, Mean ± SD			
Before intervention	12.5 ± 3.8	13.1 ± 3.5	.472
After intervention	18.2 ± 2.1	15.0 ± 2.9	<.001
Change in score	+5.7 ± 2.5	+1.9 ± 1.8	<.001

FOIS = Functional Oral Intake Scale, GUSS = Gugging Swallowing Screen, IQR = interquartile range, SD = standard deviation.

### 3.4. Evaluation of airway protection ability

Patients in the speaking valve group exhibited greater improvement in airway protection ability. After intervention, the speaking valve group showed a significantly higher peak cough flow compared with the nonspeaking valve group (168.9 ± 40.1 L/min vs 138.5 ± 36.7 L/min, *P* < .001), and their cough strength scores were also significantly better (4 [3–4] vs 3 [2–3], *P* < .001). In addition, the daily number of suctioning episodes was significantly lower in the speaking valve group (3.0 [2.0–4.0] times vs 4.5 [4.0–5.3] times, *P* < .001; Table 4).

**Table 4:** Comparison of Airway Protection Capacity Between the 2 Groups.

Outcome measure	Speaking valve group (n = 40)	Nonspealing valve group (n = 40)	*P* value
Cough peak flow (L/min), mean ± SD			
Before intervention	125.6 ± 35.2	120.8 ± 38.5	.551
After intervention	168.9 ± 40.1	138.5 ± 36.7	<.001
Semi-Quantitative Cough Strength Score (SCSS), median (IQR)			
Before intervention	2 (2–3)	2 (2–3)	.781
After intervention	4 (3–4)	3 (2–3)	<.001
Sputum production (Frequency/d), median (IQR)			
Before intervention	5.0 (4.0–6.0)	5.0 (4.0–6.0)	.892
After intervention	3.0 (2.0–4.0)	4.5 (4.0–5.3)	<.001

IQR = interquartile range, SCSS = Semi-Quantitative Cough Strength Score, SD = standard deviation.

### 3.5. Ultrasound assessment of diaphragmatic function

Diaphragmatic ultrasound results indicated more pronounced enhancement of diaphragmatic function in the speaking valve group. Although there was no significant difference in end-expiratory diaphragm thickness between the 2 groups (*P* > .05), the end-inspiratory diaphragm thickness in the speaking valve group was significantly greater than that in the nonspeaking valve group (0.41 ± 0.08 cm vs 0.35 ± 0.07 cm, *P* < .001). Furthermore, the diaphragm thickening fraction was also significantly higher in the speaking valve group (68.5 ± 15.2% vs 47.3 ± 12.8%, *P* < .001; Table 5).

**Table 5:** Comparison of diaphragmatic function assessed by ultrasonography between the 2 groups.

Diaphragmatic parameter	Speaking valve group (n = 40)	Nonspeaking valve group (n = 40)	*P* value
End-expiratory thickness (cm), mean ± SD	0.25 ± 0.06	0.24 ± 0.05	.401
End-inspiratory thickness (cm), mean ± SD	0.41 ± 0.08	0.35 ± 0.07	<.001
Diaphragmatic thickening fraction (DTF, %), mean ± SD	68.5 ± 15.2	47.3 ± 12.8	<.001

DTF = diaphragmatic thickening fraction, SD = standard deviation.

### 3.6. Respiratory physiological parameters and clinical stability

The speaking valve group demonstrated better respiratory physiological status and clinical stability. After intervention, the speaking valve group showed a higher oxygenation index (285.6 ± 45.3 mm Hg vs 250.1 ± 52.7 mm Hg, *P* = .002) and a lower respiratory rate (18.5 ± 3.2 breaths/min vs 22.4 ± 4.8 breaths/min, *P* < .001). In addition, the incidence of respiratory distress was significantly lower in the speaking valve group (5.0% [2/40] vs 22.5% [9/40], *P* = .022; Table 6).

**Table 6:** Comparison of respiratory physiological parameters and clinical stability.

Parameter	Speaking valve group (n = 40)	Nonspeaking valve group (n = 40)	*P* value
Peripheral oxygen saturation (SpO_2_, %), mean ± SD	96.8 ± 1.5	95.2 ± 2.1	<.001
Oxygenation index (PaO_2_/FiO_2_, mm Hg), mean ± SD	285.6 ± 45.3	250.1 ± 52.7	.002
Respiratory rate (breaths/min), mean ± SD	18.5 ± 3.2	22.4 ± 4.8	<.001
Heart rate (beats/min), mean ± SD	82.6 ± 9.1	88.3 ± 11.5	.015
Mean arterial pressure (MAP, mm Hg), mean ± SD	85.4 ± 7.2	83.1 ± 8.9	.194
Incidence of respiratory distress, n (%)	2 (5.0%)	9 (22.5%)	.022

MAP = Mean arterial pressure, PaO_2_/FiO_2_ = arterial oxygen pressure/inspired oxygen fraction, SD = standard deviation, SpO_2_ = peripheral oxygen saturation.

## 4. Discussion

This retrospective cohort study systematically evaluated the association between speaking valve use and decannulation outcomes and multisystem functional recovery in tracheostomized patients. After adjustment for multiple comparisons, speaking valve use was associated with a significantly shorter time to decannulation. The speaking valve group also demonstrated a higher successful decannulation rate and a lower 72-hour reintubation rate in absolute terms; however, these differences did not remain statistically significant after Bonferroni correction. In addition, speaking valve use was associated with improvements in swallowing function, airway protection, diaphragmatic performance, and respiratory physiological status.^[11]^

The reduction of approximately 5.5 days in median time to decannulation may have clinical relevance because a shorter period of tracheostomy exposure could potentially reduce tracheostomy-related burden and facilitate rehabilitation.^[12]^ Although the speaking valve group had an absolute 22.5-percentage-point higher successful decannulation rate and a 17.5-percentage-point lower 72-hour reintubation rate, these differences did not remain statistically significant after Bonferroni correction. Therefore, these findings should be interpreted as exploratory numerical trends rather than definitive evidence that speaking valve use increases decannulation success or reduces reintubation. The lack of corrected statistical significance may be related in part to the modest sample size and the conservative nature of the Bonferroni procedure.^[13]^

Regarding swallowing function, the significant improvements in FOIS and GUSS scores in the speaking valve group may stem from the device’s multifaceted physiological impact once transglottic airflow is restored.^[14]^ The restoration of subglottic pressure may help trigger pre-swallow glottic closure reflexes, thereby enhancing airway protection, while improved laryngeal sensory input may refine the timing and coordination of swallowing initiation.^[15]^ Critically, this enhancement in swallowing safety is intimately linked to airway protection – improved glottic closure during swallowing directly contributes to the patient’s ability to defend the lower airway from aspiration, forming a functional bridge between swallowing competence and cough efficacy.

The speaking valve’s beneficial effects on airway protection further reinforce this integrated response. The observed increase in peak cough flow and improved cough strength scores reflect the physiological resistance generated when expiratory airflow is directed through the glottis, effectively mimicking a more normal cough mechanism.^[16]^ This enhanced cough efficacy, together with the reduced daily frequency of suctioning, indicates an improved ability to clear secretions independently – an essential factor in preventing post-decannulation pulmonary complications.^[17]^ Effective airway clearance not only reduces infection risk but also alleviates respiratory burden, creating conditions that facilitate diaphragmatic recovery.

Diaphragmatic function, as assessed by ultrasound, provides further insight into this interconnected physiological network. The significant increase in diaphragm thickening fraction suggests enhanced diaphragmatic contractile efficiency, which may be related to the restoration of physiological end-expiratory positive pressure provided by the speaking valve.^[18]^ Appropriate levels of positive end-expiratory pressure can reduce elastic work during early inspiration, improve lung volumes, and optimize the force–length relationship of the diaphragm – an effect particularly important in patients with prolonged mechanical ventilation.^[19]^ Additionally, it is important to acknowledge that the observed improvement in diaphragmatic function is likely multifactorial in nature. Beyond the direct effect of restored expiratory resistance, enhanced diaphragm thickening may also reflect concurrent improvements in overall respiratory mechanics, reduced work of breathing, better oxygenation, and heightened clinical stability achieved through the cumulative benefits of speaking valve use across multiple physiological domains.^[19]^ Improved diaphragmatic efficiency, in turn, supports more stable breathing patterns and reduces the metabolic cost of ventilation, further reinforcing both swallowing coordination and cough effectiveness.

Regarding respiratory physiological parameters, the speaking valve group demonstrated better oxygenation, a lower respiratory rate, and reduced cardiovascular stress responses, collectively depicting a more stable respiratory state.^[20]^ The restoration of transglottic airflow may optimize ventilation–perfusion matching and improve respiratory efficiency, thereby reducing inspiratory workload – which also explains the significantly lower incidence of respiratory distress in this group.^[21]^ Stable respiratory mechanics provide the physiological foundation upon which safe swallowing, effective coughing, and sustained diaphragmatic performance depend.

Importantly, the physiological improvements observed in this study – enhanced swallowing function, improved cough effectiveness, better diaphragmatic performance, and more stable respiratory physiology – appear to occur concurrently and are functionally interdependent. These changes may be interconnected through synergistic pathways. For example, improved cough strength and airway clearance may reduce secretion retention and pneumonia risk, leading to better oxygenation and reduced respiratory workload. Improved diaphragmatic efficiency may further support respiratory stability, facilitating safer swallowing and reducing aspiration risk. Together, these interrelated effects may create a positive feedback loop that promotes overall readiness for successful decannulation.

Our findings are generally consistent with previous studies suggesting potential benefits of speaking valve use in tracheostomized patients. Eichar et al reported that speaking valve application was associated with greater readiness for tracheostomy tube removal and higher decannulation rates. Similarly, Zhang et al suggested that earlier speaking valve implementation may accelerate functional recovery. Our study adds to this literature by demonstrating a statistically significant reduction in time to decannulation after correction for multiple comparisons, together with improvements across several physiological domains. Although favorable numerical differences were observed in successful decannulation and reintubation rates, these outcomes did not remain statistically significant after Bonferroni correction and should therefore be confirmed in adequately powered prospective studies.

Nevertheless, some differences between our results and previous reports should be acknowledged. Several earlier studies and systematic reviews primarily focused on communication outcomes, patient comfort, or isolated physiological parameters, without evaluating hard clinical endpoints such as time to decannulation or reintubation. In contrast, our study used PSM to minimize confounding and incorporated multidimensional outcome assessments. Variations in patient characteristics, indications for tracheostomy, timing and duration of speaking valve use, decannulation protocols, and study methodology may also contribute to discrepancies among studies. These differences highlight the importance of standardized protocols and underscore the need for future multicenter randomized controlled trials to further clarify the magnitude of benefit and identify patient populations most likely to benefit from speaking valve therapy.

Despite the strengths of this propensity score–matched analysis, several limitations should be acknowledged. First, the retrospective design inevitably carries the risk of unmeasured confounding. Although major demographic and clinical variables were carefully balanced, some potentially relevant factors – such as specific tracheostomy techniques, cuff management strategies, variations in clinician-driven decannulation decision-making, and family support – were not fully captured. Second, this was a single-center study with a relatively modest matched sample size (80 patients), which limited subgroup analyses and may restrict generalizability. Third, the use of the speaking valve was not randomized, introducing potential selection bias whereby clinicians may have preferentially applied the device to patients perceived to have a better prognosis; PSM mitigated but could not completely eliminate this bias.^[22]^

Additionally, 2 factors related to the real-world nature of the data warrant mention. Although post-intervention assessments were standardized to occur within 24 hours prior to decannulation and at a fixed time of day, variability in the exact timing across patients was unavoidable due to differences in individual recovery trajectories and enrollment dates. Similarly, while the speaking valve protocol specified gradual extension of wearing time, daily wearing duration and progression pace varied among patients based on individual tolerance and clinical stability, with some experiencing tolerance-related interruptions. These variations reflect the inherent heterogeneity of retrospective data but do not diminish the overall findings, as all patients in the speaking valve group successfully achieved extended daytime wear prior to decannulation, ensuring that post-intervention assessments captured functional status after adequate adaptation.

The study also evaluated 3 related decannulation outcomes, requiring correction for multiple comparisons. After Bonferroni adjustment, only time to decannulation remained statistically significant, whereas the differences in successful decannulation and 72-hour reintubation were no longer significant. The Bonferroni procedure is conservative, particularly in studies with correlated outcomes and modest sample sizes, and may increase the probability of type II error. Nevertheless, the corrected results provide a more cautious estimate of the evidence and underscore the need for larger, prospectively powered studies.

Future prospective randomized controlled trials with larger, multicenter cohorts are needed to confirm these findings, explore optimal timing and patient selection, assess long-term outcomes, and evaluate cost-effectiveness.

In summary, speaking valve use was associated with a shorter time to decannulation and with improvements in swallowing function, airway protection, diaphragmatic performance, and respiratory physiological stability. Although the speaking valve group showed favorable numerical trends in successful decannulation and 72-hour reintubation rates, these differences were not statistically significant after Bonferroni correction. These findings support further prospective investigation of speaking valve therapy as a component of multidisciplinary rehabilitation for tracheostomized patients.

## Author contributions

**Conceptualization:** Minghua Yuan, Fengjun Liu, Xia Wu, Haifei Peng, Ting Hu, Xueqin Yu, Xiaoyuan Wang.

**Data curation:** Minghua Yuan, Fengjun Liu, Xia Wu, Haifei Peng, Ting Hu, Xueqin Yu, Xiaoyuan Wang.

**Formal analysis:** Minghua Yuan, Fengjun Liu, Haifei Peng, Ting Hu, Xueqin Yu, Xiaoyuan Wang.

**Investigation:** Xiaoyuan Wang.

**Funding acquisition:** Minghua Yuan, Xiaoyuan Wang.

**Writing – original draft:** Minghua Yuan, Xiaoyuan Wang.

**Writing – review & editing:** Minghua Yuan, Xia Wu, Xiaoyuan Wang.
